# Supplementary material for: Consumer Behavior Concerning Meat Consumption: Evidence from Brazil
Source: Foods. 2023 Jan 1;12(1):188. doi: 10.3390/foods12010188 (PMC9818959; doi:10.3390/foods12010188)
Supplement: Supplementary file 1 [file foods-12-00188-s001.zip › foods-2050557-supplementary.pdf]

Supplementary Material SI - Script for semi-structured interview

Sociodemographic questions		
Nº	Questions	Classifications
1	What gender do you identify?	Feminine<br>Male<br>Other<br>prefer not to identify
2	What is your age?	Up to 19 years old<br>Between 20 and 59 years old<br>more than 60 years old
3	What is your education level?	No instruction<br>Incomplete Elementary School<br>Complete primary education<br>Incomplete high school<br>Complete high school<br>Incomplete Higher Education<br>Complete Higher Education
4	City and State of residence	
Questions about behaviors		
Nº	Questions	polls
5	Você poderia comentar um pouco sobre seus hábitos de vida em geral?	Do you smoke?<br>Do you consume alcoholic beverages?<br>Do you practice physical activity?
6	Could you comment a little about your diet?	Do you always eat at home?<br>Do you go to restaurants?<br>Who chooses and buys the food you consume?<br>Who prepares your meals?<br>Food preferences: (i) sweet; (ii) bittersweet; (iii) bitter; (iv) sour
7	Why are you reducing your meat consumption?	Health?<br>Concern about environmental sustainability? FOCUS ON THIS SURVEY<br>Concern about animal welfare/suffering? (Do you have pets?) FOCUS ON THIS SURVEY<br>Influences or recommendations? (Doctor, friend, family, co-workers, Internet, book, religion…)
8		

	Could you tell me a little about your meat reduction process from the beginning to now?	Are you reducing meats in general or just a specific type of meat?<br>What was meat consumption like before?<br>How long have you been reducing?<br>How much was reduced?<br>How often do you eat meat these days?<br>Do you want to reduce it even further?
9	How did the people around you react to the fact that you were cutting back on meat, and how did you feel about it?	Did you get support or criticism? From who? Family, friendship?
10	Has reducing meat consumption caused changes in your life or routine? What has changed?	Shopping locations?<br>Food expenses? FOCUS ON THIS SURVEY<br>Inclusion of new foods? FOCUS ON THIS SURVEY<br>Consumption of meat substitutes?
11	Do you have face-to-face or virtual contact with others who are also cutting back on meat consumption?	Who are these people, and what is the form of contact? Personal, virtual? Social networks?
12	Have you found difficulties in the process of reducing meat consumption? What were or have been the most significant difficulties for you?	
13	What could hinder those who want or need to reduce meat consumption?	
14	Do you believe that something made the meat reduction process easier for you, such as a characteristic of you or your life context?	Know how to cook?<br>Contact with other reducers?<br>Medical need?
15	What do you think could make reducing meat consumption easier for those who want or need to reduce meat as well?	Could some kind of product help in this process?
16	Would you like to make any more comments?	
